# UNDERSTANDING ENGAGEMENT OF OLDER ADULTS IN SOCIAL CONVERSATIONS TO MITIGATE SOCIAL ISOLATION AND LONELINESS

**DOI:** 10.1093/geroni/igae098.0674

**Published:** 2024-12-31

**Authors:** W Quin Yow, Ka Lon Sou, Alina Clarise Wong, Chi Shuen Ang

**Affiliations:** Singapore University of Technology & Design, Singapore, Singapore; Singapore University of Technology & Design, Singapore, Singapore; Singapore University of Technology & Design, Singapore, Singapore; Singapore University of Technology & Design, Singapore, Singapore

## Abstract

Six percent of older adults in Singapore experience social isolation. Given that social isolation and loneliness are associated with cognitive decline and increased mortality rates, there is a pressing need for effective interventions to address these issues among older adults. Traditional approaches, such as in-person befriending interventions, have encountered challenges, e.g., inflexible visit schedules and transportation barriers. There are recent interests exploring how technology can help tackle social isolation and loneliness among older adults. As part of such initiative, we develop an older-friendly conversation agent (CA) utilizing state-of-the-art large language model technology. This CA aims to provide a virtual companion to older adults while prioritizing safety, autonomy, and flexibility. To our knowledge, there are very few studies, and none in Asia, that examine how social conversations among older adults can alleviate isolation and loneliness. As a first step in this investigation, an observational study was undertaken to identify typical conversation topics among older adult friends to build the CA capability. Preliminary findings from three recorded conversations, each lasting approximately 30 minutes and involving a total of eleven older adults (aged 65-79, nine females), revealed recurring topics such as “activities”, “characteristics or personality of others and self”, “location of place or an event”, “episodic events”, “life stories”, and “surroundings”. These insights set the baseline to test the effectiveness of the technology in reducing social isolation in older adults. How interactions with the virtual CA could alleviate social isolation and loneliness among older adults will be shared at the symposium.

